# Identification of the targets of hematoporphyrin derivative in lung adenocarcinoma using integrated network analysis

**DOI:** 10.1186/s40659-019-0213-z

**Published:** 2019-02-04

**Authors:** Hongtao Yin, Yan Yu

**Affiliations:** 1 Department of Radiation Oncology, Harbin Medical University Cancer Hospital, Harbin, 150081 Heilongjiang China; 2 Department of Medical Oncology, Harbin Medical University Cancer Hospital, No. 150 Haping Road, Nangang District, Harbin, 150081 Heilongjiang China

**Keywords:** Lung adenocarcinoma, Hematoporphyrin derivative, X-ray, Protein–protein interaction network, Integrated network

## Abstract

**Background:**

Hematoporphyrin derivative (HPD) has a sensibilization effect in lung adenocarcinoma. This study was conducted to identify the target genes of HPD in lung adenocarcinoma.

**Methods:**

RNA sequencing was performed using the lung adenocarcinoma cell line A549 after no treatment or treatment with X-ray or X-ray + HPD. The differentially expressed genes (DEGs) were screened using Mfuzz package by noise-robust soft clustering analysis. Enrichment analysis was carried out using “BioCloud” online tool. Protein–protein interaction (PPI) network and module analyses were performed using Cytoscape software. Using WebGestalt tool and integrated transcription factor platform (ITFP), microRNA target and transcription factor (TF) target pairs were separately predicted. An integrated regulatory network was visualized with Cytoscape software.

**Results:**

A total of 815 DEGs in the gene set G1 (continuously dysregulated genes along with changes in processing conditions [untreated—treated with X-ray—X-ray + treated with HPD]) and 464 DEGs in the gene set G2 (significantly dysregulated between X-ray + HPD-treated group and untreated/X-ray-treated group) were screened. The significant module identified from the PPI network for gene set G1 showed that ribosomal protein L3 (*RPL3*) gene could interact with heat shock protein 90 kDa alpha, class A member 1 (*HSP90AA1*). TFs AAA domain containing 2 (ATAD2) and protein inhibitor of activated STAT 1 (PIAS1) were separately predicted for the genes in gene set G1 and G2, respectively. In the integrated network for gene set G2, ubiquitin-specific peptidase 25 (*USP25*) was targeted by *miR*-*200b*, *miR*-*200c*, and *miR*-*429*.

**Conclusion:**

*RPL3*, *HSP90AA1*, *ATAD2*, and *PIAS1* as well as *USP25,* which is targeted by *miR*-*200b*, *miR*-*200c*, and *miR*-*429*, may be the potential targets of HPD in lung adenocarcinoma.

## Background

Lung/pulmonary adenocarcinoma, a common type of lung cancer, possesses some malignant characteristics such as duct formation, gland formation, and excessive production of mucus [1]. Although smoking is responsible for most lung adenocarcinoma cases, lung adenocarcinoma is also very common in passive smokers or people with irregular smoking habit [2, 3]. In the United States, approximately 40% cases of lung cancer are lung adenocarcinoma, which often originates from the peripheral lung tissue [4] probably because the filters in cigarettes prevent the entry of the bulky grains into the lungs [5]. Although adenocarcinoma produces smaller masses and develops slowly as compared with the other types of lung cancers, it is likely to show distant metastasis at an early stage [6]. Therefore, evaluation of the mechanisms underlying lung adenocarcinoma progression is desirable to improve treatment outcomes.

Porphyrins may selective accumulate in malignant tumors [7], and hematoporphyrin derivative (HPD) has been used as a photosensitizer in the photodynamic therapy (PDT) of lung cancer [8]. It was demonstrated that 99mTc-hematoporphyrin-linked albumin nanoparticles (99mTc-HP-ANPs) may be used for the PDT and radio-diagnosis of lung cancer [9]. The combination of PDT and photosensitizers HPD and 5-aminolevulinic acid (ALA) may increase the curative rate for skin cancers, distinctly cut down the photosensitive period, and reduce the dose of pro-toxic HPD [10]. Hematoporphyrin conjugated with the antibodies directed to vascular endothelial growth factor shows high antitumor activities in patients with Lewis lung carcinoma [11]. The expression level of interleukin-6 affects the cellular sensitivity to PDT, and the combination of PDT and interleukin-6 may serve as a novel strategy for the therapy of Lewis lung carcinoma [12]. Wang et al. used X-ray as the energy source for PDT activation and suggested that the X-ray-induced photodynamic therapy (X-PDT) may be used as a novel therapeutic method against human cancers [13]. Austerlitz et al. found that the response of the Fricke solution doped with hematoporphyrin and irradiated with low-energy X-rays was enhanced in PDT [14]. However, the mechanism underlying HPD effects in lung adenocarcinoma are not investigated.

As a commonly studied lung adenocarcinoma cell line, A549 was used as a model in the present study. RNA sequencing was applied to the untreated A549 cells as well as those treated with X-ray or the combination of X-ray and HPD. The differentially expressed genes (DEGs) were screened and enrichment analysis, protein–protein interaction (PPI) network, and module analyses as well as integrated network analysis were carried out to identify the important genes affected by HPD.

## Materials and methods

### Cell cultivation

The lung adenocarcinoma cell line A549 was acquired from the Cell Bank of Chinese Academy of Sciences. The cells were cultured in Dulbecco’s modified Eagle’s medium (DMEM) (GIBCO) supplemented with 1% penicillin/streptomycin double-antibody (GIBCO) and 10% fetal bovine serum (FBS, GIBCO) in a humidified 5% CO_2_ incubator (Thermo) at 37 °C. After the medium was discarded, the cells were washed once with cold phosphate-buffered saline (PBS) and treated with pancreatin (GIBCO) at 37 °C for 2 min. The suspension was treated with complete medium to neutralize pancreatin, and the mixture was centrifuged (1000 rpm, 5 min). The supernatant was discarded and the cells were resuspended in FBS-supplemented DMEM and cultured in a humidified 5% CO_2_ incubator (Thermo) at 37 °C.

### Cell counting kit-8 (CCK-8) assay

The cells were counted, seeded into 96-well plates (ABI, 1 × 10^4^ cells/well), and cultured in a humidified 5% CO_2_ incubator (Thermo) at 37 °C overnight. Following incubation, cells were treated with different concentrations of HPD (0, 2.5, 5, 10, 20, 30, 60, and 120 μg/mL) and different doses of X-ray (0, 5, 10, and 15 Gy; dose rate was 1 Gy/min) for 24 h. After treatment, the cells were incubated with CCK-8 solution (Tongren; 10 μL/well) at 37 °C for 1 h. The absorbance value of each well was measured with BIV-TEK INSTRUMENTS INC (TECAN) at an optical density (OD) of 450 nm wavelength, and the cell proliferation activity was calculated.

### Flow cytometry assay

To detect the apoptosis of A549 cells, flow cytometry assay was conducted according to the previously described method [15]. Cells were counted and cultured into six-well plates (ABI, 2 × 10^5^ cells/well) in a humidified 5% CO_2_ incubator (Thermo) at 37 °C overnight. The cells were subsequently treated with different concentrations of HPD and different doses of X-ray. After the medium was discarded, the cells were digested with pancreatin (GIBCO), followed by treatment with fresh medium to deactivate pancreatin and centrifugation (1000 rpm, 6 min). The supernatant was discarded and the cells were washed once with PBS and resuspended in 1× binding buffer (BD Biosciences; 400 μL 1× binding buffer for the control group and 100 μL 1× binding buffer for other groups). A total of 100 μL of the above solution was transferred into flow tubes and treated with 5 μL of fluorescein isothiocyanate (FITC)-Annexin V (BD Biosciences) and 5 μL of propidium iodide (PI, BD Biosciences, 50 μg/mL) (the control group was divided into unstained, Annexin V-stained, PI-stained, and Annexin V + PI-stained groups). After being incubated (in the absence of light) for 15 min at room temperature, the cells were treated with 400 μL of 1× binding buffer (BD Biosciences) and analyzed with a flow cytometer (BD Biosciences).

### RNA extraction and RNA-seq library construction

The cells were counted, seeded into 100 mm culture dishes (1 × 10^6^ cells/well), and cultured in a humidified 5% CO_2_ incubator (Thermo) at 37 °C overnight. After treatment with different concentrations of HPD or different doses of X-ray (the control group was left untreated; the X-ray group was treated with 10 Gy X-ray and cultured for 24 h; X-ray + HPD group was treated with 10 Gy X-ray + 10 μg/mL of HPD and cultured for 24 h; each group had three replicates), the cells were washed twice with cold PBS. Total RNA was extracted using Trizol reagent (TaKaRa) following the manufacturer’s instruction and quantified with a spectrophotometer (Nanodrop). RNA-seq library was constructed with NEBNext^®^ Ultra™ RNA Library Prep Kit for Illumina^®^ (New England Biolabs) and sequencing was performed on Illumina Hiseq 4000 (PE150) (Illumina). The sequencing data were deposited into the Sequence Read Archive (SRA) database under the accession number of SRP091521.

### Data preprocessing and DEG screening

The Prinseq-lite (http://edwards.sdsu.edu/cgi-bin/prinseq/prinseq.cgi) tool [16] and FASTX_ToolKit (http://hannonlab.cshl.edu/fastx_toolkit/) [17] were applied for the quality control of the raw data. Barcode and adaptor sequences in the reads were removed. The reads with N content larger than 5% were filtered out. Bases with continuous quality under 10 at 5′ or 3′ end were discarded. The reads with low quality (having over 20% bases with quality lower than 20) and those shorter than 30 nt were removed. The clean reads obtained from the three groups of samples were mapped to GRCH38 human genome using TopHat software (version 2.0.8) [18]. The fragments per kilobase million (FPKM) and read count matrix of the genes were acquired using StringTie tool [19], and gene annotation information was obtained from GENCODE database (version 24, http://genome.imim.es/gencode/) [20]. To identify DEGs, noise-robust soft clustering analysis was performed using the fuzzy c-means clustering algorithm in Mfuzz package (http://www.bioconductor.org/packages/release/bioc/html/Mfuzz.html) [21]. Specific clusters were selected based on gene expression trends. Both minSTD and acore parameters were set as 0.5.

### Functional and pathway enrichment analysis

Gene ontology (GO, http://www.geneontology.org) database can use structured vocabularies for noting genes or gene products from three aspects (MF, molecular function; BP, biological process; and CC, cellular component) [22]. The Kyoto Encyclopedia of Genes and Genomes (KEGG, http://www.genome.ad.jp/kegg) database links genomic information with functional information through the investigation of gene functions [23]. “BioCloud” online tool (http://www.biocloudservice.com) is developed for settling computing problems of high-throughput biological data. Using “BioCloud” online tool, DEGs were subjected to GO functional and KEGG pathway enrichment analyses with the threshold of *p*-value < 0.05.

### PPI network and module analyses

Search Tool for the Retrieval of Interacting Genes (STRING, http://string-db.org/) is a database that collects the PPIs involving more than 1100 organisms [24]. Based on STRING database [24], the PPIs among the proteins corresponding to DEGs were analyzed with a combined score > 0.4 as the cut-off criterion. PPI network was subsequently visualized using Cytoscape software (http://www.cytoscape.org) [25], and the hub nodes [26] in the PPI network were screened by calculating their connectivity degrees. Module analysis for PPI network was conducted using the Molecular Complex Detection (MCODE) plugin [27] of Cytoscape software. In addition, enrichment analysis was performed for the nodes of significant modules using “BioCloud” online tool.

### Integrated network analysis

WEB-based gene set analysis toolkit (WebGestalt, http://www.webgestalt.org) [28] was used to predict the genes involved in the PPI network at *p* < 0.001 and the number of target genes ≥ 4 as the thresholds. Using Cytoscape software [25], the microRNA (miRNA) target regulatory network was constructed. According to the integrated transcription factor platform (ITFP, http://itfp.biosino.org/itfp) [29], the transcription factors (TFs) targeting DEGs and the differentially expressed TFs were predicted. The TF target regulatory network was visualized with Cytoscape software [25]. The PPI network, miRNA target regulatory network, and TF target regulatory network were integrated, and an integrated network was constructed with Cytoscape software [25].

### Statistical analysis

One-way analysis of variance and two-tailed *t*-test were applied for statistical analysis using GraphPad prism software (GraphPad Software, San Diego, CA). Data were shown as the mean ± standard error of the mean (SEM). A value of *p* < 0.05 was considered statistically significant.

## Results

### Effects of HPD and X-ray on the proliferation and apoptosis of A549 cells

**Fig. 1:**
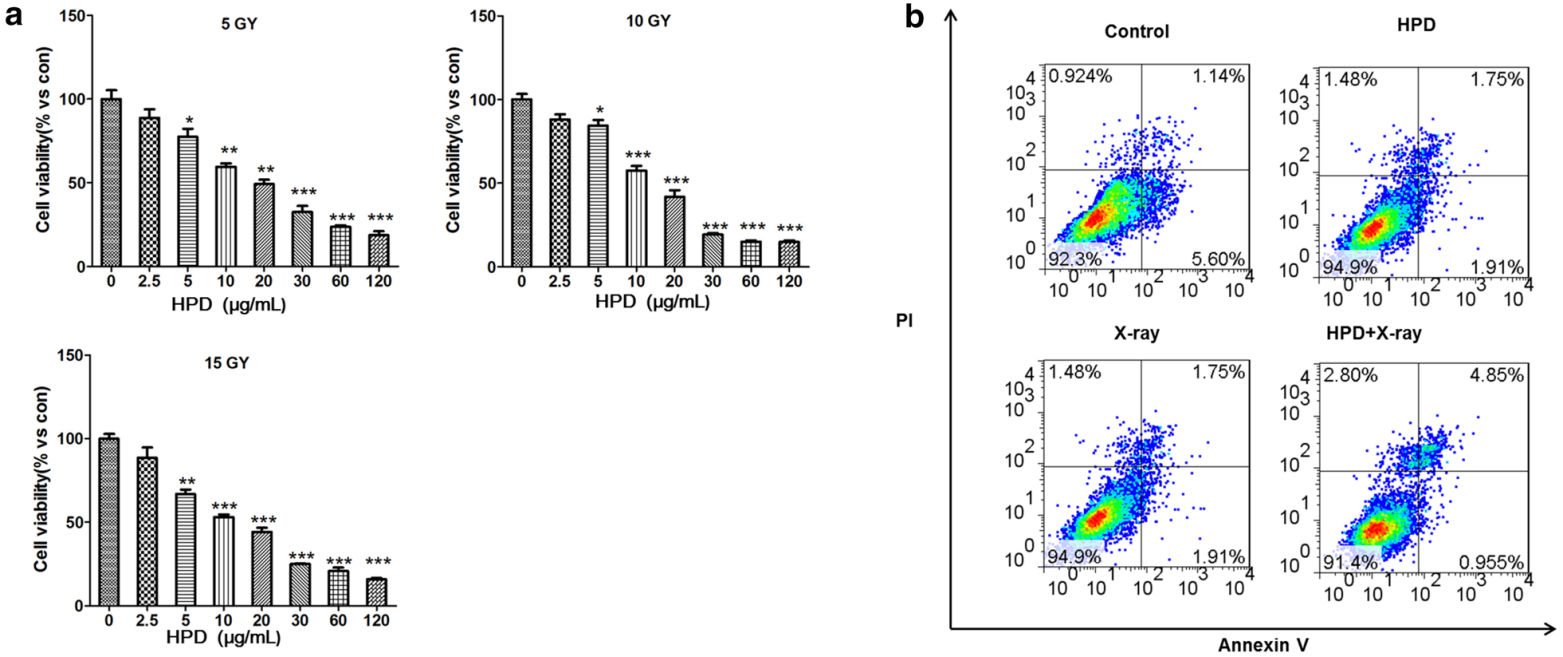
Effects of the combination of HPD and X-ray. The combination significantly suppressed the proliferation activity (**a**) and promoted apoptosis of A549 cells (HPD: 10 μg/mL; X-ray: 10 Gy irradiation) (**b**). *HPD* hematoporphyrin derivative, *PI* propidium iodide

A549 cells were treated with different concentrations of HPD and different doses of X-ray and their proliferation rate was calculated. HPD in combination with X-ray significantly suppressed the proliferation rate of A549 cells (*p* < 0.05, Fig. 1a). In particular, 10 μg/mL of HPD and 10 Gy X-ray was the lowest concentration-dose combination that showed significant effects on A549 cell proliferation (*p* < 0.001). A549 cells were treated with 10 μg/mL of HPD and 10 Gy X-ray, and their apoptosis rate was analyzed with flow cytometry. The results of flow cytometry analysis showed that the combination of HPD (10 μg/mL) and X-ray (10 Gy) significantly increased the apoptosis of A549 cells (*p* < 0.05, Fig. 1b). Therefore, the combination of 10 μg/mL of HPD and 10 Gy X-ray was used in the subsequent experiments.

### Data preprocessing and DEG analysis

**Fig. 2:**
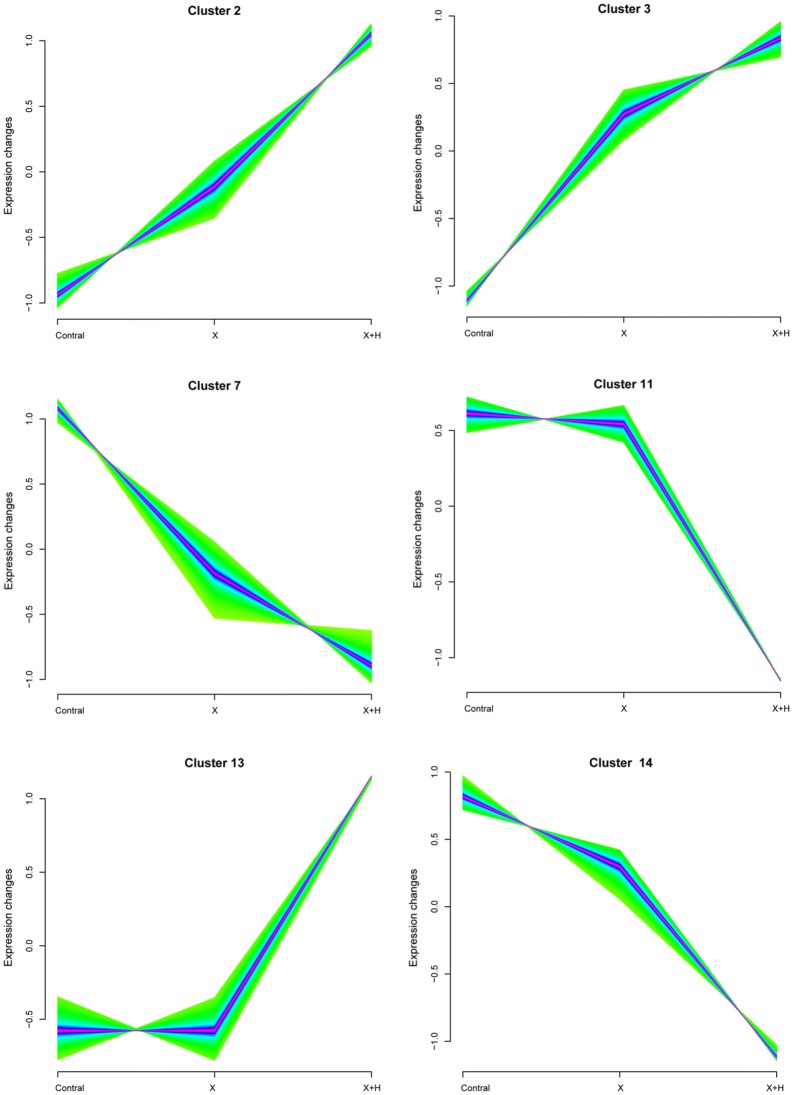
The clusters 2, 3, 7, 11, 13, and 14 obtained from soft clustering analysis

Data sequencing was carried out with quality control (Table 1), and the sequences were mapped to GRCH38 human genome (Table 2). The gene expression matrix was processed with Mfuzz package to reveal a total of 14 clusters (Fig. 2). According to the experimental design, only two types of clusters were selected for analysis. One type of clusters showed continuous upregulation (cluster 2 and 3) or downregulation (cluster 7 and 14) of gene expression along with the change in processing conditions (untreated—treated with X-ray—treated with X-ray + HPD) (containing a total of 815 genes that were included in gene set G1). Another type of cluster included the significantly upregulated (cluster 13) or downregulated (cluster 11) genes under the processing condition of X-ray + HPD in comparison with the processing condition of untreated and X-ray treatment (containing a total of 464 genes that were included in gene set G2).

**Table 1:** The results of quality control for sequencing data

Sample	Raw reads	Raw base	Clean read	Clean base	Clean reads rate (%)	Clean reads mean length
C1_R1	23,719,333	3,557,899,950	22,042,930	2,960,605,850	92.93	134.3109038
C1_R2	23,719,333	3,557,899,950	19,989,260	2,662,625,066	84.27	133.2027832
C2_R1	34,224,122	5,133,618,300	32,282,452	4,221,477,560	94.33	130.766943
C2_R2	34,224,122	5,133,618,300	30,870,743	4,017,646,477	90.20	130.1441458
C3_R3	28,305,868	4,245,880,200	26,491,724	3,503,162,645	93.59	132.2361144
C3_R3	28,305,868	4,245,880,200	25,565,624	3,364,967,177	90.32	131.6207724
H1_R1	31,013,406	4,652,010,900	29,028,783	3,809,754,255	93.60	131.2405778
H1_R2	31,013,406	4,652,010,900	28,211,189	3,695,864,424	90.96	131.0070421
H2_R1	36,876,370	5,531,455,500	34,583,327	4,464,527,411	93.78	129.0947922
H2_R2	36,876,370	5,531,455,500	33,557,059	4,323,966,213	91.00	128.854147
H3_R1	31,602,257	4,740,338,550	29,790,898	3,839,171,450	94.27	128.8706185
H3_R2	31,602,257	4,740,338,550	28,994,009	3,728,257,975	91.75	128.5871842
X1_R1	38,506,430	5,775,964,500	36,323,284	4,706,649,914	94.33	129.5766626
X1_R2	38,506,430	5,775,964,500	35,392,852	4,573,185,587	91.91	129.2121242
X2_R1	26,676,689	4,001,503,350	24,977,453	3,294,932,070	93.63	131.9,162,554
X2_R2	26,676,689	4,001,503,350	23,234,815	3,048,373,656	87.10	131.1985336
X3_R1	26,125,305	3,918,795,750	24,664,499	3,184,364,078	94.41	129.1071867
X3_R2	26,125,305	3,918,795,750	23,447,530	3,013,771,864	89.75	128.5325944

**Table 2:** The results of mapping the sequencing data to human genome

Sample name	Left		Right		All pair	
	Mapped	Uniquely mapped	Mapped	Uniquely mapped	Mapped	Uniquely mapped
C1	20,710,400	18,614,230	17,931,401	16,062,833	2,123,508	1,629,067
C2	29,825,454	26,711,264	27,530,725	24,622,143	3,273,719	2,485,977
C3	24,351,734	22,204,068	23,021,298	21,001,313	2,555,418	1,941,532
X1	33,144,667	29,801,821	31,766,989	28,555,706	3,845,887	2,915,483
X2	23,108,910	20,910,087	20,617,622	18,640,706	2,428,431	1,848,198
X3	22,680,212	20,131,394	20,793,708	18,409,380	2,466,001	1,866,161
H1	26,599,811	23,805,716	25,426,744	22,751,212	2,999,459	2,248,238
H2	31,741,919	28,699,363	30,212,029	27,324,878	3,534,230	2,607,919
H3	27,165,798	24,935,225	25,977,523	23,876,280	2,940,041	2,153,962

### Functional and pathway enrichment analysis

**Fig. 3:**
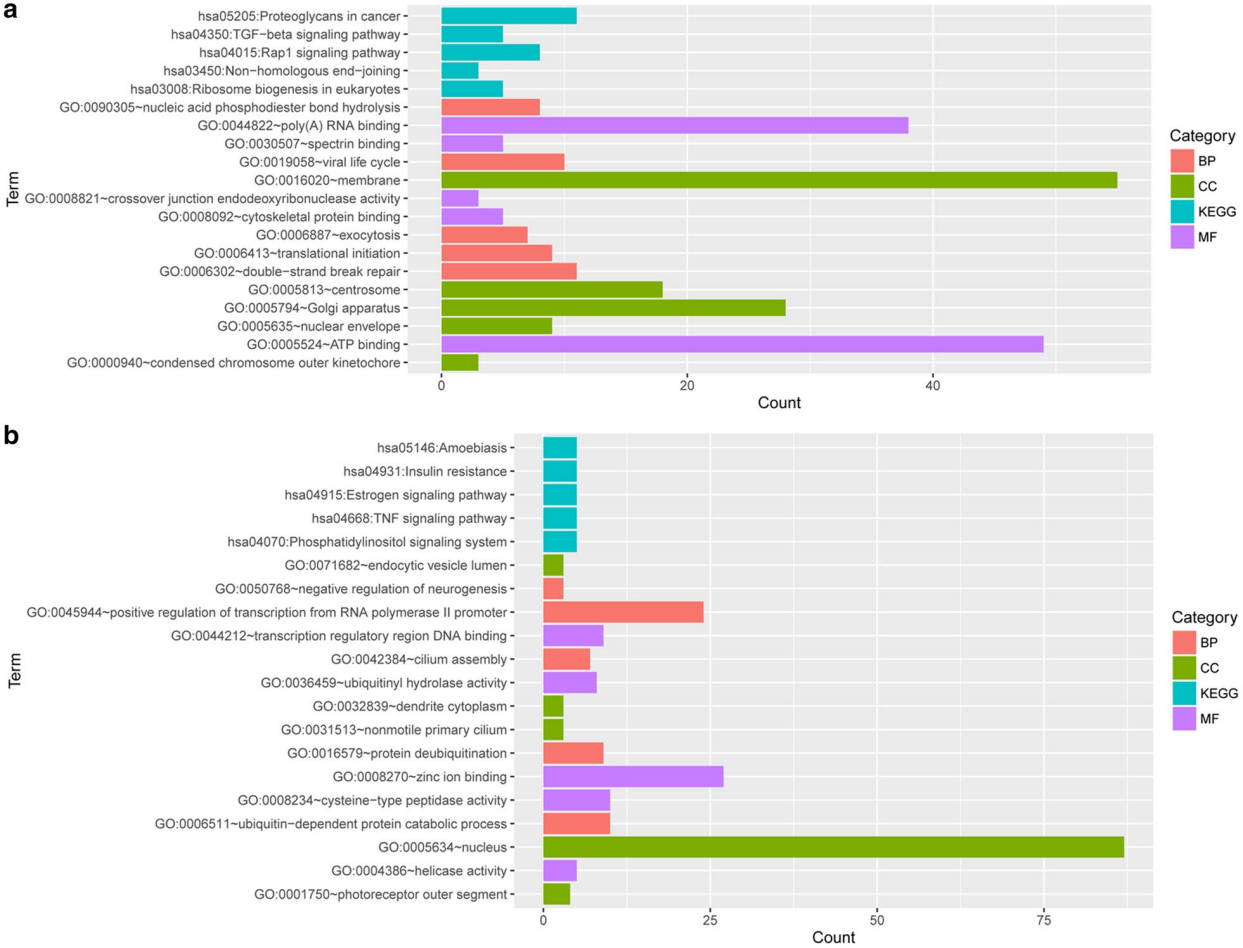
The top five enriched terms. **a** Genes in gene set G1 and **b** genes in gene set G2. *BP* biological process, *CC* cellular component, *MF* molecular function, *KEGG* Kyoto Encyclopedia of Genes and Genomes

The genes in gene set G1 and G2 were separately subjected to functional and pathway enrichment analyzes. The top five enriched terms in BP, CC, MF, and KEGG categories are shown in Fig. 3. The genes in gene set G1 were mainly enriched in double-strand break repair (BP), membrane (CC), ATP binding (MF), and proteoglycans in cancer (KEGG) (Fig. 3a). The genes in gene set G2 were mainly enriched in positive regulation of transcription from RNA polymerase II promoter (BP), nucleus (CC), zinc ion binding (MF), and estrogen signaling pathway (KEGG) (Fig. 3b).

### PPI network and module analyses

**Fig. 4:**
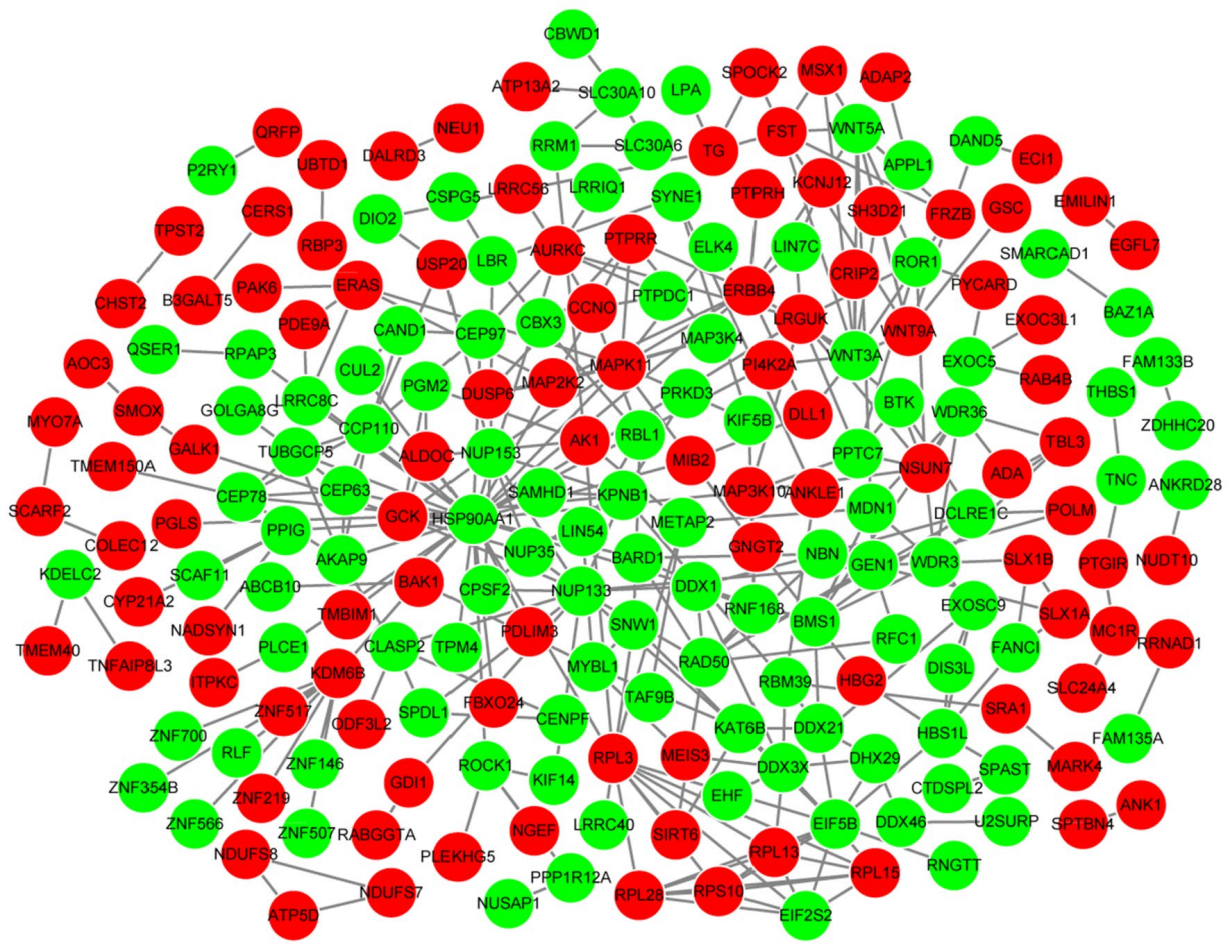
The protein–protein interaction network constructed for the genes in gene set G1. Red and green circles represent the upregulated and downregulated genes, respectively

**Fig. 5:**
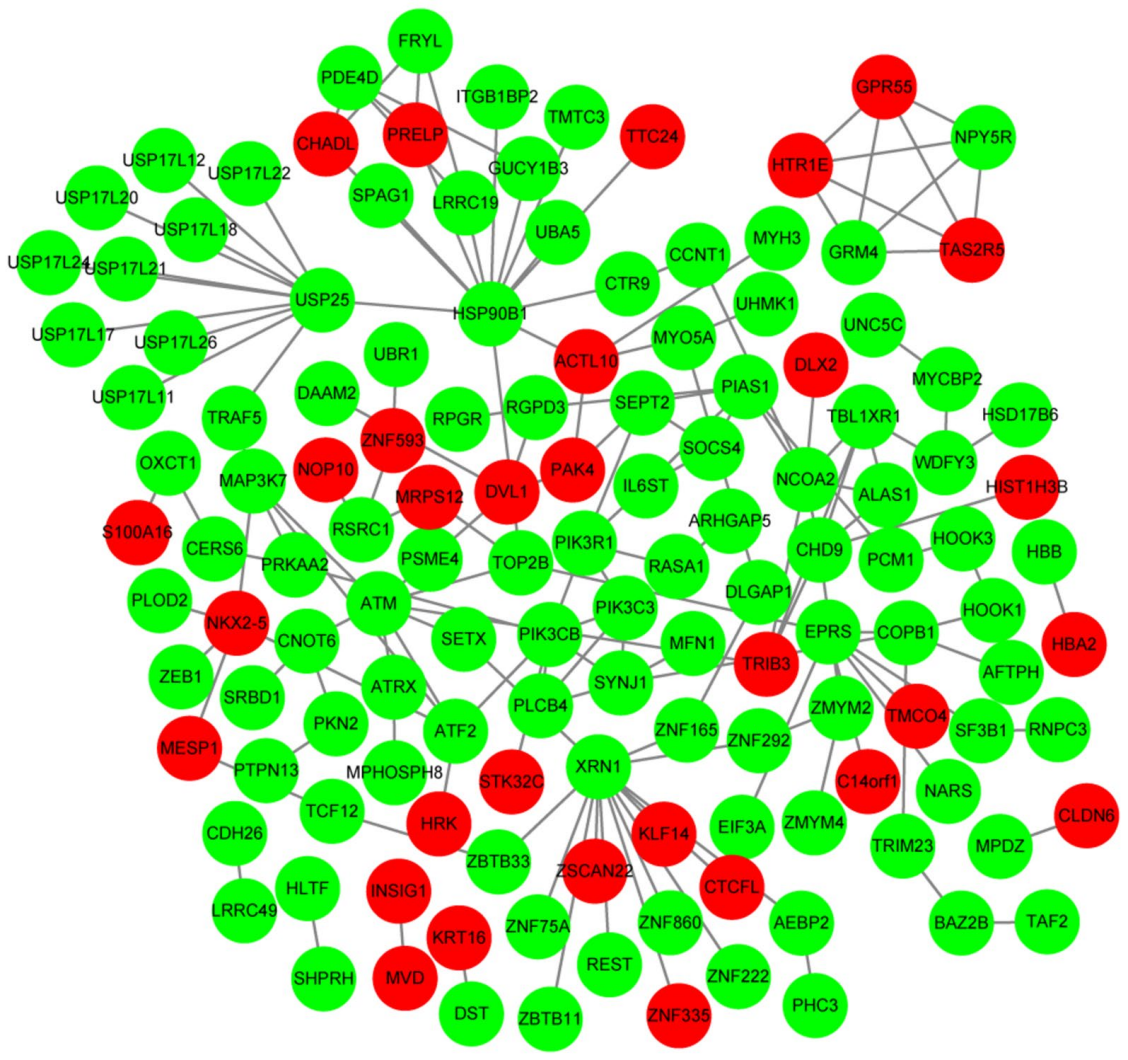
The protein–protein interaction network constructed for the genes in gene set G2. Red and green circles represent the upregulated and downregulated genes, respectively

**Fig. 6:**
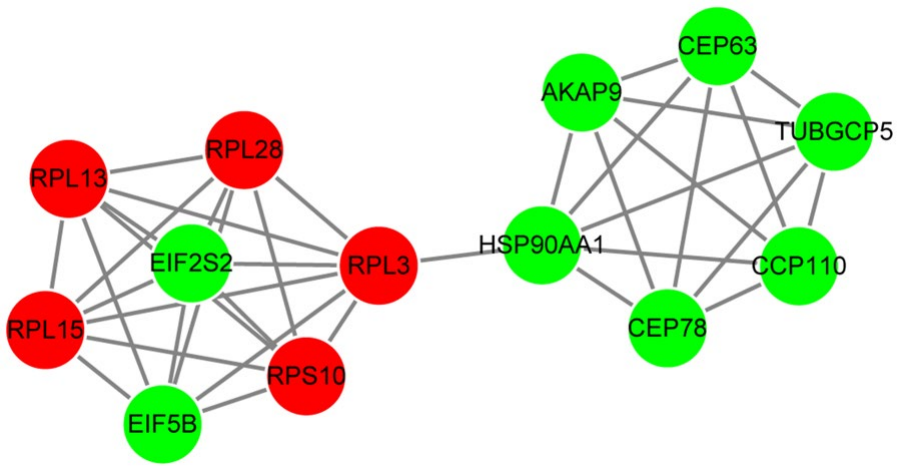
The significant module identified from the protein–protein interaction network constructed for the genes in gene set G1. Red and green circles represent the upregulated and downregulated genes, respectively

The PPI network constructed for the genes in gene set G1 had 210 nodes and 333 interactions (Fig. 4). On the other hand, the PPI network constructed for the genes in gene set G2 had 135 nodes and 164 interactions (Fig. 5). The top 10 nodes with high degrees in PPI networks are listed in Table 3 and included heat shock protein 90 kDa alpha, class A member 1 (*HSP90AA1*, degree = 31) and ribosomal protein L3 (*RPL3*, degree = 10). Based on MCODE plugin, one significant module was identified from the PPI network constructed for the genes in gene set G1 that included 13 nodes and 37 interactions (such as *HSP90AA1*-*RPL3*) (Fig. 6). The terms enriched for the genes in the module are listed in Table 4, and mainly included translational initiation (BP, *p*-value = translational initiation), cytosol (CC, *p*-value = 1.28E−09), poly(A) RNA binding (MF, *p*-value = 1.70E−06), and ribosome (KEGG, *p*-value = 4.57E−06). No significant module was identified from the PPI network constructed for the genes in gene set G2.

**Table 3:** The top 10 nodes in the protein–protein interaction networks constructed for the genes in gene set G1 and the genes in gene set G2

Gene set	Gene	Degree
Gene set G1	HSP90AA1	31
	NUP133	13
	EIF5B	12
	MAPK11	11
	ERBB4	10
	WNT3A	10
	RPL3	10
	DDX3X	9
	KDM6B	9
	GCK	9
Gene set G2	XRN1	14
	HSP90B1	13
	USP25	11
	ATM	8
	EPRS	7
	PIK3CB	7
	NCOA2	7
	CHD9	6
	PIAS1	6
	PIK3R1	5

**Table 4:** The terms enriched for the genes in the significant module (only listed terms with *p*-value < 0.01)

Category	Term	Count	P-value	Gene symbol
BP	GO:0006413 ~ translational initiation	7	2.35E−10	*RPL13*, *RPL15*, *EIF2S2*, *RPL3*, *EIF5B*, *RPS10*, *RPL28*
BP	GO:0006412 ~ translation	7	2.20E−08	*RPL13*, *RPL15*, *EIF2S2*, *RPL3*, *EIF5B*, *RPS10*, *RPL28*
BP	GO:0000086 ~ G2/M transition of mitotic cell cycle	6	3.17E−08	*HSP90AA1*, *TUBGCP5*, *CCP110*, *CEP78*, *AKAP9*, *CEP63*
BP	GO:0006415 ~ translational termination	5	3.21E−07	*RPL13*, *RPL15*, *RPL3*, *RPS10*, *RPL28*
BP	GO:0016259 ~ selenocysteine metabolic process	5	3.70E−07	*RPL13*, *RPL15*, *RPL3*, *RPS10*, *RPL28*
BP	GO:0006414 ~ translational elongation	5	5.47E−07	*RPL13*, *RPL15*, *RPL3*, *RPS10*, *RPL28*
BP	GO:0006614 ~ SRP-dependent cotranslational protein targeting to membrane	5	8.12E−07	*RPL13*, *RPL15*, *RPL3*, *RPS10*, *RPL28*
BP	GO:0019083 ~ viral transcription	5	9.41E−07	*RPL13*, *RPL15*, *RPL3*, *RPS10*, *RPL28*
BP	GO:0001887 ~ selenium compound metabolic process	5	1.01E−06	*RPL13*, *RPL15*, *RPL3*, *RPS10*, *RPL28*
BP	GO:0000184 ~ nuclear-transcribed mRNA catabolic process, nonsense-mediated decay	5	1.20E−06	*RPL13*, *RPL15*, *RPL3*, *RPS10*, *RPL28*
BP	GO:0019058 ~ viral life cycle	5	3.22E−06	*RPL13*, *RPL15*, *RPL3*, *RPS10*, *RPL28*
BP	GO:0000278 ~ mitotic cell cycle	6	8.02E−06	*HSP90AA1*, *TUBGCP5*, *CCP110*, *CEP78*, *AKAP9*, *CEP63*
BP	GO:0044267 ~ cellular protein metabolic process	7	1.67E−05	*RPL13*, *RPL15*, *EIF2S2*, *RPL3*, *EIF5B*, *RPS10*, *RPL28*
BP	GO:0010467 ~ gene expression	7	2.65E−05	*RPL13*, *RPL15*, *EIF2S2*, *RPL3*, *EIF5B*, *RPS10*, *RPL28*
BP	GO:0006996 ~ organelle organization	5	4.52E−05	*HSP90AA1*, *CCP110*, *CEP78*, *AKAP9*, *CEP63*
BP	GO:0034641 ~ cellular nitrogen compound metabolic process	5	6.51E−05	*RPL13*, *RPL15*, *RPL3*, *RPS10*, *RPL28*
BP	GO:0016032 ~ viral process	5	4.78E−04	*RPL13*, *RPL15*, *RPL3*, *RPS10*, *RPL28*
BP	GO:0044281 ~ small molecule metabolic process	6	5.11E−03	*HSP90AA1*, *RPL13*, *RPL15*, *RPL3*, *RPS10*, *RPL28*
CC	GO:0005829 ~ cytosol	13	1.28E−09	*HSP90AA1*, *TUBGCP5*, *CCP110*, *RPL13*, *CEP78*, *RPL15*, *EIF2S2*, *RPL3*, *EIF5B*, *RPS10*, *AKAP9*, *CEP63*, *RPL28*
CC	GO:0022625 ~ cytosolic large ribosomal subunit	4	7.57E−06	*RPL13*, *RPL15*, *RPL3*, *RPL28*
CC	GO:0005813 ~ centrosome	5	1.28E−04	*TUBGCP5*, *CCP110*, *CEP78*, *AKAP9*, *CEP63*
CC	GO:0016020 ~ membrane	5	3.24E−02	*HSP90AA1*, *RPL13*, *RPL15*, *RPS10*, *RPL28*
CC	GO:0005840 ~ ribosome	2	3.80E−02	*RPL15*, *RPS10*
MF	GO:0044822 ~ poly(A) RNA binding	8	1.70E−06	*HSP90AA1*, *RPL13*, *RPL15*, *EIF2S2*, *RPL3*, *EIF5B*, *RPS10*, *RPL28*
MF	GO:0003735 ~ structural constituent of ribosome	5	7.88E−06	*RPL13*, *RPL15*, *RPL3*, *RPS10*, *RPL28*
MF	GO:0003723 ~ RNA binding	5	1.47E−04	*RPL13*, *RPL15*, *EIF2S2*, *RPL3*, *RPL28*
MF	GO:0005515 ~ protein binding	11	9.64E−03	*HSP90AA1*, *CCP110*, *RPL13*, *RPL15*, *EIF2S2*, *RPL3*, *EIF5B*, *RPS10*, *AKAP9, CEP63, RPL28*
KEGG	hsa03010 ~ ribosome	5	4.57E−06	*RPL13*, *RPL15*, *RPL3*, *RPS10*, *RPL28*

*BP* biological process, *CC* cellular component, *MF* molecular function, *KEGG* Kyoto Encyclopedia of Genes and Genomes

### Integrated network analysis

**Fig. 7:**
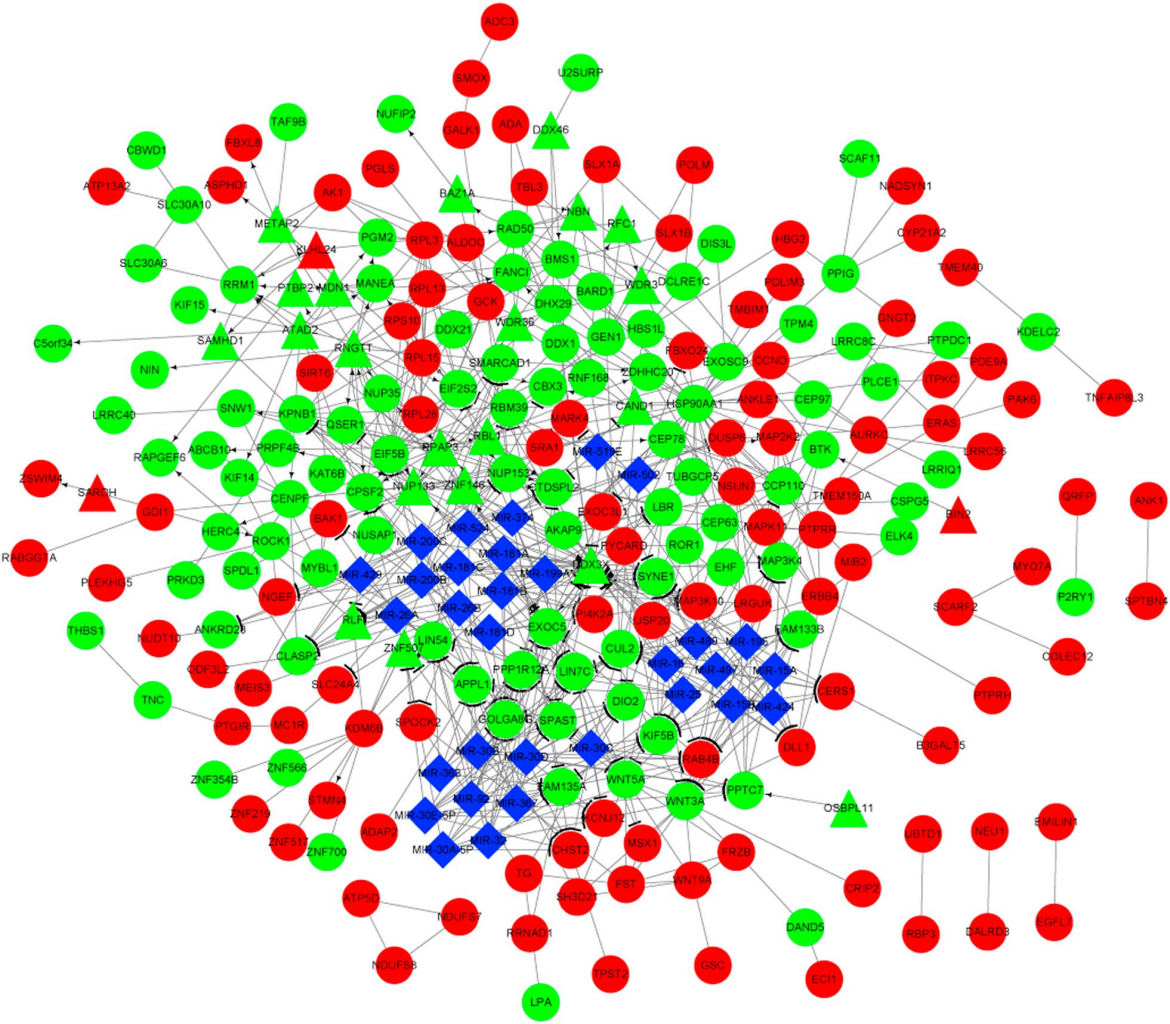
The integrated network constructed for the genes in gene set G1. Red and green separately indicate the upregulated and downregulated genes, respectively. Circles and triangles represent genes and transcription factors, respectively. Blue diamonds indicate miRNAs

**Fig. 8:**
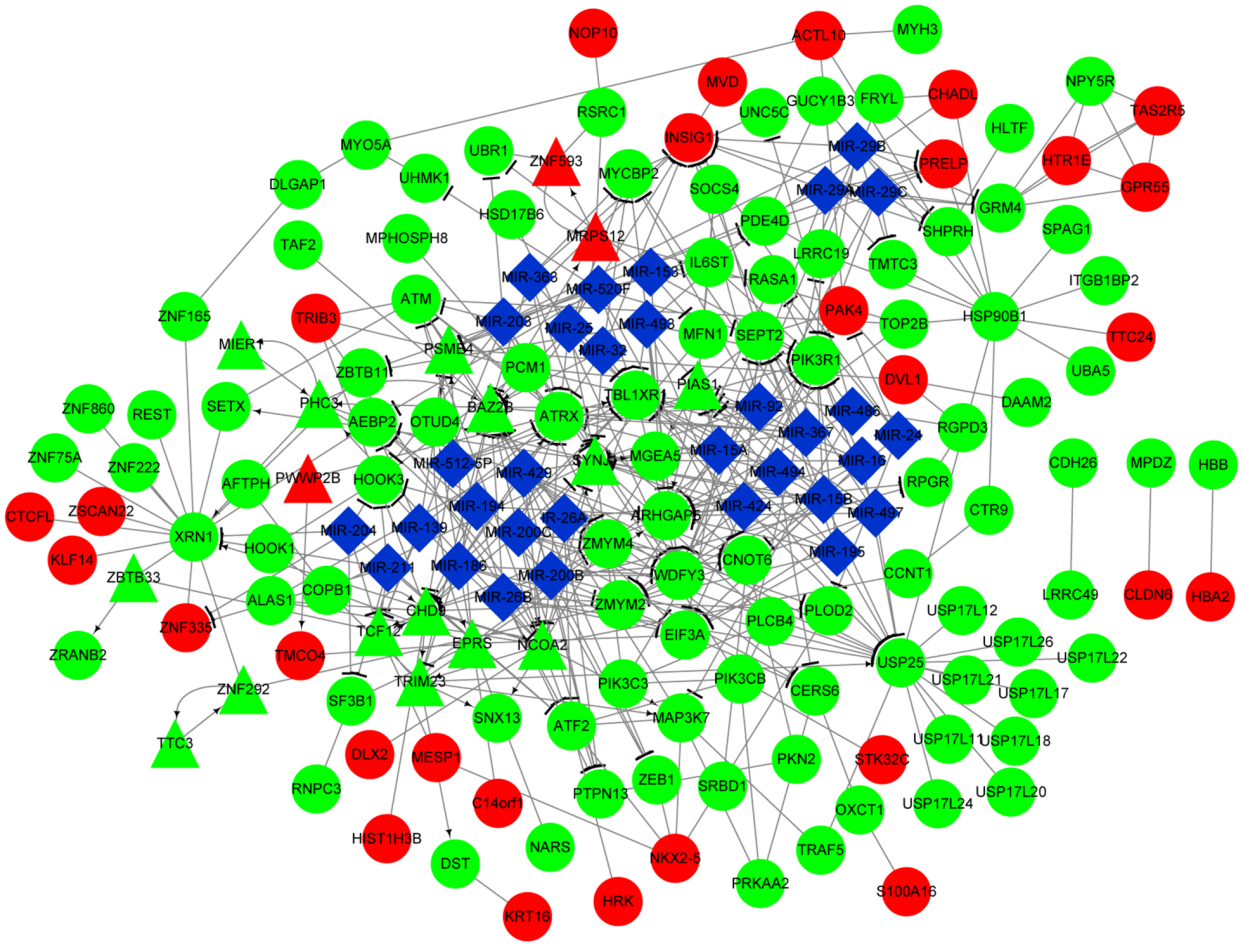
The integrated network constructed for the genes in gene set G2. Red and green separately indicate the upregulated and downregulated genes, respectively. Circles and triangles indicate genes and transcription factors, respectively. Blue diamonds indicate miRNAs

The miRNAs of the genes implicated in the PPI networks constructed for the genes in gene set G1 (Table 5) and G2 (Table 6) were predicted. The TFs targeting the genes in gene set G1 (ATPase family, AAA domain containing 2 [*ATAD2*]) and G2 (protein inhibitor of activated STAT 1 [*PIAS1*]) were also analyzed (Table 7). An integrated network was constructed for the genes in gene set G1 that had 259 nodes (including 25 TFs and 31 miRNAs) and 687 pairs (Fig. 7). The integrated network for the genes in gene set G2 was also visualized; it carried 174 nodes (including 18 TFs and 32 miRNAs) and 445 pairs (Fig. 8). In particular, ubiquitin-specific peptidase 25 (*USP25*) was targeted by *miR*-*200b*, *miR*-*200c*, and *miR*-*429* in the integrated network for the genes in gene set G2. The top 30 nodes with high degrees in the integrated networks are listed in Table 8.

**Table 5:** The miRNAs targeted the genes implicated in the protein–protein interaction networks constructed for the genes in gene set G1

microRNA	Count	Statistics
hsa_TATTATA, MIR-374	9	*p*-value = 1.15e−05
hsa_CAGTATT, MIR-200B, MIR-200C, MIR-429	11	*p*-value = 1.92e−05
hsa_GTGCAAT, MIR-25, MIR-32, MIR-92, MIR-363, MIR-367	8	*p*-value = 1.00e−04
hsa_CTTTGTA, MIR-524	9	*p*-value = 0.0003
hsa_GGCACTT, MIR-519E	5	*p*-value = 0.0003
hsa_CTACTGT, MIR-199A	6	*p*-value = 0.0003
hsa_GCAAGGA, MIR-502	4	*p*-value = 0.0004
hsa_TGTTTAC, MIR-30A-5P, MIR-30C, MIR-30D, MIR-30B, MIR-30E-5P	10	*p*-value = 0.0005
hsa_TGAATGT, MIR-181A, MIR-181B, MIR-181C, MIR-181D	9	*p*-value = 0.0006
hsa_TGCTGCT, MIR-15A, MIR-16, MIR-15B, MIR-195, MIR-424, MIR-497	10	*p*-value = 0.0007
hsa_TACTTGA, MIR-26A, MIR-26B	7	*p*-value = 0.0007
hsa_ATGTCAC, MIR-489	4	*p*-value = 0.0008

**Table 6:** The miRNAs targeted the genes implicated in the protein–protein interaction networks constructed for the genes in gene set G2

microRNA	Count	Statistics
hsa_CAGTATT, MIR-200B, MIR-200C, MIR-429	13	*p*-value = 1.27e-09
hsa_CATTTCA, MIR-203	10	*p*-value = 1.29e-08
hsa_TACTTGA, MIR-26A, MIR-26B	8	*p*-value = 2.86e-06
hsa_ATGTACA, MIR-493	8	*p*-value = 4.11e-06
hsa_AAGCACT, MIR-520F	7	*p*-value = 6.53e-06
hsa_ATTCTTT, MIR-186	7	*p*-value = 1.56e-05
hsa_GCTGAGT, MIR-512-5P	4	*p*-value = 1.69e-05
hsa_GTACAGG, MIR-486	4	*p*-value = 2.43e-05
hsa_AAAGGGA, MIR-204, MIR-211	6	*p*-value = 4.87e-05
hsa_TGCTGCT, MIR-15A, MIR-16, MIR-15B, MIR-195, MIR-424, MIR-497	9	*p*-value = 6.55e-05
hsa_TGGTGCT, MIR-29A, MIR-29B, MIR-29C	8	*p*-value = 1.00e-04
hsa_ATGTTTC, MIR-494	5	*p*-value = 1.00e-04
hsa_GTGCAAT, MIR-25, MIR-32, MIR-92, MIR-363, MIR-367	6	*p*-value = 0.0003
hsa_CTGTTAC, MIR-194	4	*p*-value = 0.0003
hsa_CTATGCA, MIR-153	5	*p*-value = 0.0004
hsa_ACTGTAG, MIR-139	4	*p*-value = 0.0005
hsa_CTGAGCC, MIR-24	5	*p*-value = 0.0006

**Table 7:** The transcription factors (TFs) targeting the genes in gene set G1 and gene set G2

G1 gene list		G2 gene list	
TF	Count	TF	Count
*RNGTT*	14	*TRIM23*	7
*RPAP3*	13	*NCOA2*	6
*ATAD2*	10	*PHC3*	4
*ZNF146*	6	*SYNJ1*	4
*NUP133*	5	*CHD9*	3
*METAP2*	4	*PIAS1*	3
*RBL1*	4	*BAZ2B*	2
*DDX3X*	3	*EPRS*	2
*KLHL24*	3	*MRPS12*	2
*PTBP2*	3	*PSME4*	2
*BAZ1A*	2	*PWWP2B*	2
*CAND1*	2	*MIER1*	1
*MDN1*	2	*TCF12*	1
*SARDH*	2	*TTC3*	1
*WDR36*	2	*ZBTB33*	1
*BIN2*	1	*ZNF292*	1
*DDX46*	1	*ZNF593*	1
*NBN*	1		
*OSBPL11*	1		
*RFC1*	1		
*RLF*	1		
*SAMHD1*	1		
*WDR3*	1		
*ZNF507*	1

**Table 8:** The top 30 nodes with higher degrees in the integrated networks for gene set G1 and gene set G2

G1 gene list		G2 gene list	
Node	Degree	Node	Degree
*DDX3X*	34	*SYNJ1*	25
*HSP90AA1*	31	*USP25*	23
*NUP133*	21	*TBL1XR1*	21
*RPAP3*	19	*NCOA2*	20
*RNGTT*	17	*PIK3R1*	18
*WNT3A*	16	*XRN1*	18
*SYNE1*	15	*CHD9*	17
*EIF5B*	15	*BAZ2B*	17
*GOLGA8G*	14	*PIAS1*	16
*KIF5B*	14	*ATRX*	14
*EXOC5*	14	*ARHGAP5*	14
*WNT5A*	13	*MIR*-*200B*	13
*PPP1R12A*	13	*MIR*-*200C*	13
*CUL2*	13	*MIR*-*429*	13
*LIN7C*	13	*HSP90B1*	13
*NUP153*	12	*WDFY3*	12
*RAB4B*	12	*TRIM23*	12
*MIR*-*200B*	11	*INSIG1*	11
*CTDSPL2*	11	*ATM*	11
*RLF*	11	*SEPT2*	11
*APPL1*	11	*MIR*-*203*	10
*MIR*-*200C*	11	*PSME4*	10
*MIR*-*429*	11	*EIF3A*	10
*PPTC7*	11	*ZMYM2*	10
*MIR*-*30B*	11	*EPRS*	10
*KPNB1*	11	*CNOT6*	9
*MAPK11*	11	*ATF2*	9
*EIF2S2*	10	*MIR*-*15A*	9
*CLASP2*	10	*MIR*-*16*	9
*DLL1*	10	*MIR*-*15B*	9

## Discussion

In this study, the CCK-8 assay result revealed the significant suppression in the proliferation activity of A549 cells in response to the treatment with the combination of HPD and X-ray. The combination of 10 μg/mL of HPD and 10 Gy X-ray was selected as the lowest concentration/dose that achieved a significant increase in the apoptosis of A549 cells, which might be a limitation in terms of analysis of the data. In Europe, hematoporphyrin is the most commonly used photosensitizer for the treatment of advanced lung cancer [30, 31]. Previous studies have demonstrated that HPD-PDT may inhibit proliferation and induce apoptosis of A549 cells, thereby inducing effective killing of adenocarcinoma cells [32, 33]. After the optimization of the combination treatment of HPD and X-ray, a series of bioinformatic analyses were performed with the RNA-seq data.

Through noise-robust soft clustering analysis, 815 genes that showed continuous upregulated or downregulated expression along with the change in processing conditions (untreated—treated with X-ray—treated with X-ray + HPD) were included in the gene set G1. A total of 464 genes that were significantly upregulated or downregulated under the processing condition of X-ray + HPD were included in the gene set G2. The significant module identified from the PPI network constructed for the genes in gene set G1 revealed the interaction between *RPL3* and *HSP90AA1*. RPL3 functions in the response of cells to oxaliplatin- and 5-fluorouracil-induced nucleolar stress and may be used to improve the therapeutic effects of these drugs against cancers [34]. The chemotherapy curative effect of actinomycin D is determined by *RPL3* status in cancers shorting of *p53*; thus, high level of *RPL3* may be useful for the treatment of lung and colon cancers [35]. The frequencies of mutant genotypes of *HSP90AA1*, *HSP90AB1*, and *HSP90B1* are reported to be significantly higher in the patients with non-small cell lung cancer (NSCLC) in the Turkish population [36]. Downregulation of HSP90 expression correlated with increased overall survival of patients with NSCLC, and HSP90 inhibitor exerts an antiproliferative effect on NSCLC cell lines [37, 38]. These observations suggest that *RPL3* interacting with *HSP90AA1* may be associated with the sensibilization effect of HPD in lung adenocarcinoma.

*ATAD2* and *PIAS1* were separately predicted as the TFs targeting the genes from the gene sets G1 and G2. Caron et al. demonstrated that ATAD2 overexpression may promote the malignant transformation of lung and breast cancers by affecting the basic properties of chromatin [39]. Wang et al. found that ATAD2/AAA^+^ nuclear coregulatory cancer associated (*ANCCA*) may serve as a promising biomarker for the treatment and prognosis of squamous cell lung carcinoma [40]. PIAS1 contributes to cytoplasm-nuclear distribution of focal adhesion kinase by interacting with it, and focal adhesion kinase activity in the nucleus facilitates survival and progression of NSCLC via promotion of DNA repair regulation and cell-extracellular matrix interaction [41, 42]. *PIAS1* mediates oncogenic signaling by promoting promyelocytic leukemia (PML) degradation, and PIAS1 and PML expression is negatively correlated in NSCLC cell lines [43]. Therefore, *ATAD2* and *PIAS1* may be involved in the action mechanism of HPD in lung adenocarcinoma.

In the integrated network for the genes in the gene set G2, *USP25* was targeted by *miR*-*200b*, *miR*-*200c*, and *miR*-*429*. *miR*-*200c* may serve as a tumor suppressor in NSCLC through the inhibition of *USP25* expression and may be applied for therapeutic purposes [44]. The overexpression of *miR*-*200c* and *miR*-*141* is associated with the short overall survival of patients with lung adenocarcinoma via angiogenesis and mesenchymal-epithelial transition [45]. The low expression of *miR*-*200b* is reported to induce E2F transcription factor 3 overexpression and increase the chemoresistance of patients with lung adenocarcinoma to docetaxel [46]. Zhu et al. suggested that the serum levels of *miR*-*29c* and *miR*-*429* may be used as non-invasive biomarkers for patients with early stage NSCLC [47]. Lang et al. found that *miR*-*429* contributes to cell proliferation and metastasis and regulates several tumor suppressor genes in patients with NSCLC, serving as a possible therapeutic target [48]. These observations suggest that *USP25* targeted by *miR*-*200b*, *miR*-*200c*, and *miR*-*429* may also function in the action process of HPD in lung adenocarcinoma.

## Conclusion

A total of 815 DEGs in gene set G1 were identified along with a change in processing conditions (untreated—treated with X-ray—treated with X-ray + HPD). In addition, 464 DEGs in gene set G2 were screened under the processing condition of X-ray + HPD. *RPL3*, *HSP90AA1*, *ATAD2*, as well as *PIAS1* and *USP25,* which is targeted by *miR*-*200b*, *miR*-*200c*, and *miR*-*429* may show correlations with the sensibilization effect of HPD in lung adenocarcinoma. Further validation with experimental research is warranted to confirm the roles of these genes in the sensibilization effect of HPD in lung adenocarcinoma.

## Highlights

1. In the significant module for gene set G1, *RPL3* could interact with *HSP90AA1*.
2. *ATAD2* and *PIAS1* were the transcription factors separately targeting the gene set G1 and G2.
3. In the integrated network, *miR-200b*, *miR-200c*, and *miR-429* co-regulated *USP25*.

## Abbreviations

HPD: hematoporphyrin derivative
DEGs: differentially expressed genes
PPI: protein–protein interaction
ITFP: integrated transcription factor platform
TF: transcription factor
PDT: photodynamic therapy
X-PDT: X-ray-induced photodynamic therapy
DMEM: Dulbecco’s modified Eagle’s medium
PBS: phosphate-buffered saline
CCK-8: cell counting kit-8
FITC: fluorescein isothiocyanate
FPKM: fragments per kilobase million
