# Correction to: Identification of the targets of hematoporphyrin derivative in lung adenocarcinoma using integrated network analysis

**DOI:** 10.1186/s40659-020-00281-8

**Published:** 2020-04-19

**Authors:** Hongtao Yin, Yan Yu

**Affiliations:** 1 Department of Radiation Oncology, Harbin Medical University Cancer Hospital, Harbin, 150081 Heilongjiang China; 2 Department of Medical Oncology, Harbin Medical University Cancer Hospital, No. 150 Haping Road, Nangang District, Harbin, 150081 Heilongjiang China

## Correction to: Biol Res (2019) 52:4 10.1186/s40659-019-0213-z

The Fig. 1b is wrongly published in the original publication of the article [1]. The correct version of Fig. 1b is presented in this Correction.

**Fig. 1:**
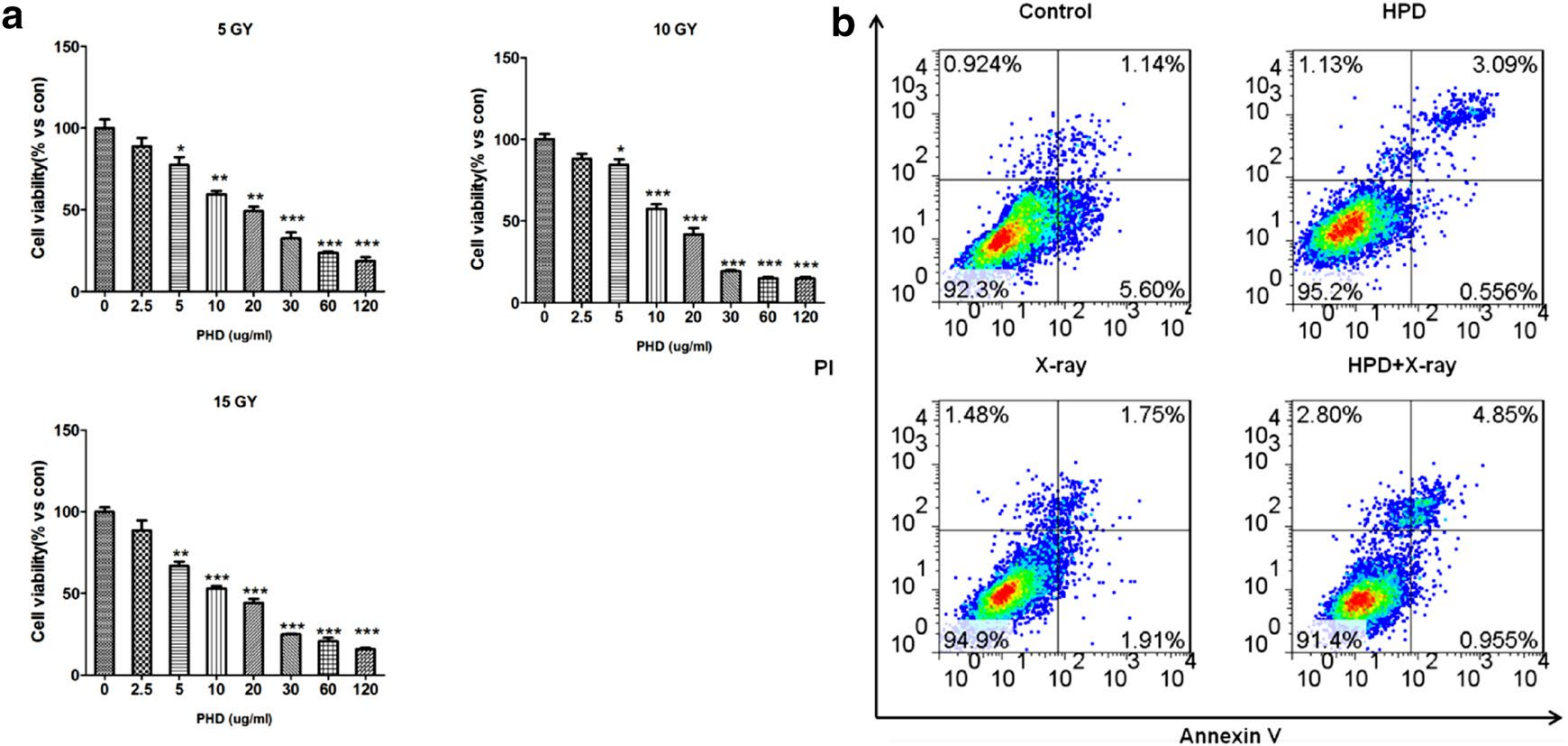
Effects of the combination of HPD and X-ray. The combination significantly suppressed the proliferation activity (**a**) and promoted apoptosis of A549 cells (HPD: 10 μg/mL; X-ray: 10 Gy irradiation) (**b**). *HPD* hematoporphyrin derivative, *PI* propidium iodide
